# *QuickStats*: Death Rates* for Motor-Vehicle–Traffic Injuries, Suicide, and Homicide Among Adolescents and Young Adults Aged 15–24 Years — United States, 1999–2019

**DOI:** 10.15585/mmwr.mm7005a6

**Published:** 2021-02-05

**Figure:**
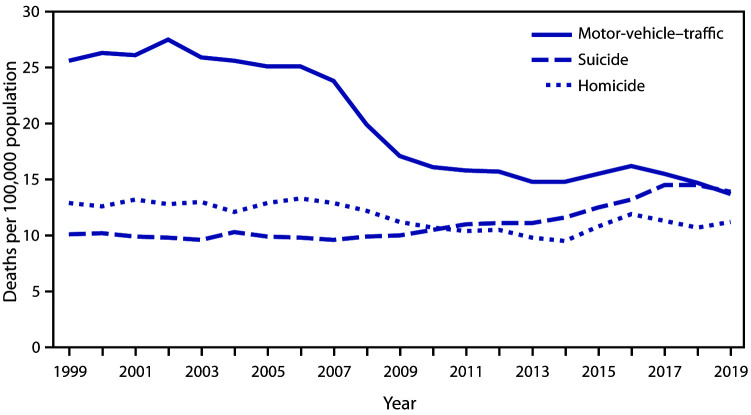
Mortality rates for adolescents and young adults aged 15–24 years for deaths from motor-vehicle–traffic injury, suicide, and homicide remained relatively stable during 1999–2006 and then exhibited different patterns through 2019. In 1999, the rate for motor-vehicle–traffic deaths was 25.6 per 100,000 population and declined to 13.7 in 2019. The suicide rate was 10.1 in 1999 and increased to 14.5 in 2018 before declining to 13.9 in 2019. The homicide rate was 12.9 in 1999 and declined to 9.5 in 2014 before increasing to 11.2 in 2019. In 2019, the death rates for motor-vehicle–traffic injury and suicide were similar; both rates were higher than the homicide rate.

**Source:** National Center for Health Statistics, National Vital Statistics System, Mortality Data, 2009–2019. https://www.cdc.gov/nchs/nvss/deaths.htm

For more information on this topic, CDC recommends the following link: https://www.cdc.gov/injury/

